# Metabolic Complications in Cardiac Aging

**DOI:** 10.3389/fphys.2021.669497

**Published:** 2021-04-29

**Authors:** Thomas Sithara, Konstantinos Drosatos

**Affiliations:** Center for Translational Medicine, Lewis Katz School of Medicine at Temple University, Philadelphia, PA, United States

**Keywords:** cardiac aging, fatty acid oxidation, carbohydrate metabolism, ketone bodies, autophagy, mitochondria

## Abstract

Aging is a process that can be accompanied by molecular and cellular alterations that compromise cardiac function. Although other metabolic disorders with increased prevalence in aged populations, such as diabetes mellitus, dyslipidemia, and hypertension, are associated with cardiovascular complications; aging-related cardiomyopathy has some unique features. Healthy hearts oxidize fatty acids, glucose, lactate, ketone bodies, and amino acids for producing energy. Under physiological conditions, cardiac mitochondria use fatty acids and carbohydrate mainly to generate ATP, 70% of which is derived from fatty acid oxidation (FAO). However, relative contribution of nutrients in ATP synthesis is altered in the aging heart with glucose oxidation increasing at the expense of FAO. Cardiac aging is also associated with impairment of mitochondrial abundance and function, resulting in accumulation of reactive oxygen species (ROS) and activation of oxidant signaling that eventually leads to further mitochondrial damage and aggravation of cardiac function. This review summarizes the main components of pathophysiology of cardiac aging, which pertain to cardiac metabolism, mitochondrial function, and systemic metabolic changes that affect cardiac function.

## Introduction

Cardiac aging is an intrinsic process, accompanied by structural and functional changes of the heart, which increase vulnerability to stress and cardiovascular-related mortality and morbidity. The most common features of cardiac aging include progressive degeneration of myocardium, impaired cardiac conduction system, aortic valvular stenosis and mitral valvular insufficiency, increased wall thickening, and stiffening of the arteries. Aging-related cardiovascular complications also include hypertension, atrial fibrillation, and atherosclerosis.

Aged myocardium presents with left ventricular hypertrophy, increased left ventricular end-diastolic pressure, lower fractional shortening, diastolic dysfunction, fibrosis, cardiomyocyte apoptosis, oxidative stress, inflammation, and lower exercise capacity (Dai et al., 2012). Age-related cardiac complications that lead to heart failure with preserved ejection fraction (HFpEF) have higher prevalence in women than men (Merz and Cheng, 2016). This difference has been attributed to differential dependence of cardiac function on estrogen signaling, which attenuates in post-menopausal women (Mahmoodzadeh et al., 2006).

Many studies have explored the association of cardiac complications with alterations in metabolic pathways (Lopaschuk, 2017). As the world population grows older and cardiovascular complications become more frequent, the involvement of alterations in cardiac metabolism in cardiac aging pathophysiology is warranted. The objective of this article pertains to better understanding of metabolic amendments that occur during cardiac aging including intermediary metabolism and mitochondrial function and may unravel novel factors that account for pathophysiology in aged hearts.

## Cardiac Metabolism and Function in the Healthy Heart

A healthy heart pumps about 7,000 litters of blood per day, which requires a significant amount of ATP that is acquired through oxidation of fatty acids, glucose, amino acids, ketone bodies, and lactate. Fatty acid oxidation (FAO) is the primary source for cardiac ATP synthesis (Lopaschuk et al., 2010). Depending on cardiac work load, hormonal status, fasting vs. feeding state and availability of energy substrates, and the ratio of energy supply from different substrates is adjusted accordingly (Ramos-Roman et al., 2012; Viscarra and Ortiz, 2013; Karwi et al., 2018).

Lipoprotein- or adipose tissue-derived free fatty acids are transported into the heart *via* the transmembrane fatty acid transporter, cluster of differentiation (CD36). For the uptake of lipids from triglyceride-rich lipoprotein, lipoprotein lipase-mediated lipolysis precedes, while whole lipoprotein uptake is also possible (Coburn et al., 2000; Bharadwaj et al., 2010; Son et al., 2018). Fatty acids are then converted to fatty acyl-CoA by acyl-CoA synthase and fatty acylcarnitine by carnitine palmitoyltransferase I (CPT-I)β (Tomec and Hoppel, 1975), which is transported into the mitochondrial matrix, where it is converted back to long-chain acyl-CoA by carnitine palmitoyltransferase II (CPT-II; Hoppel et al., 1998). β-oxidation processes fatty acyl-CoAs to acetyl-CoA yielding NADH and FADH2. Further acetyl-CoA oxidation within the mitochondrial matrix by the tricarboxylic acid cycle (TCA cycle or Krebs cycle) produces three NADHs and one FADH2 and GTP. The NADH and FADH2 are further oxidized by electron transport chain, which leads to ATP generation (Ma and Li, 2015).

**Figure 1:**
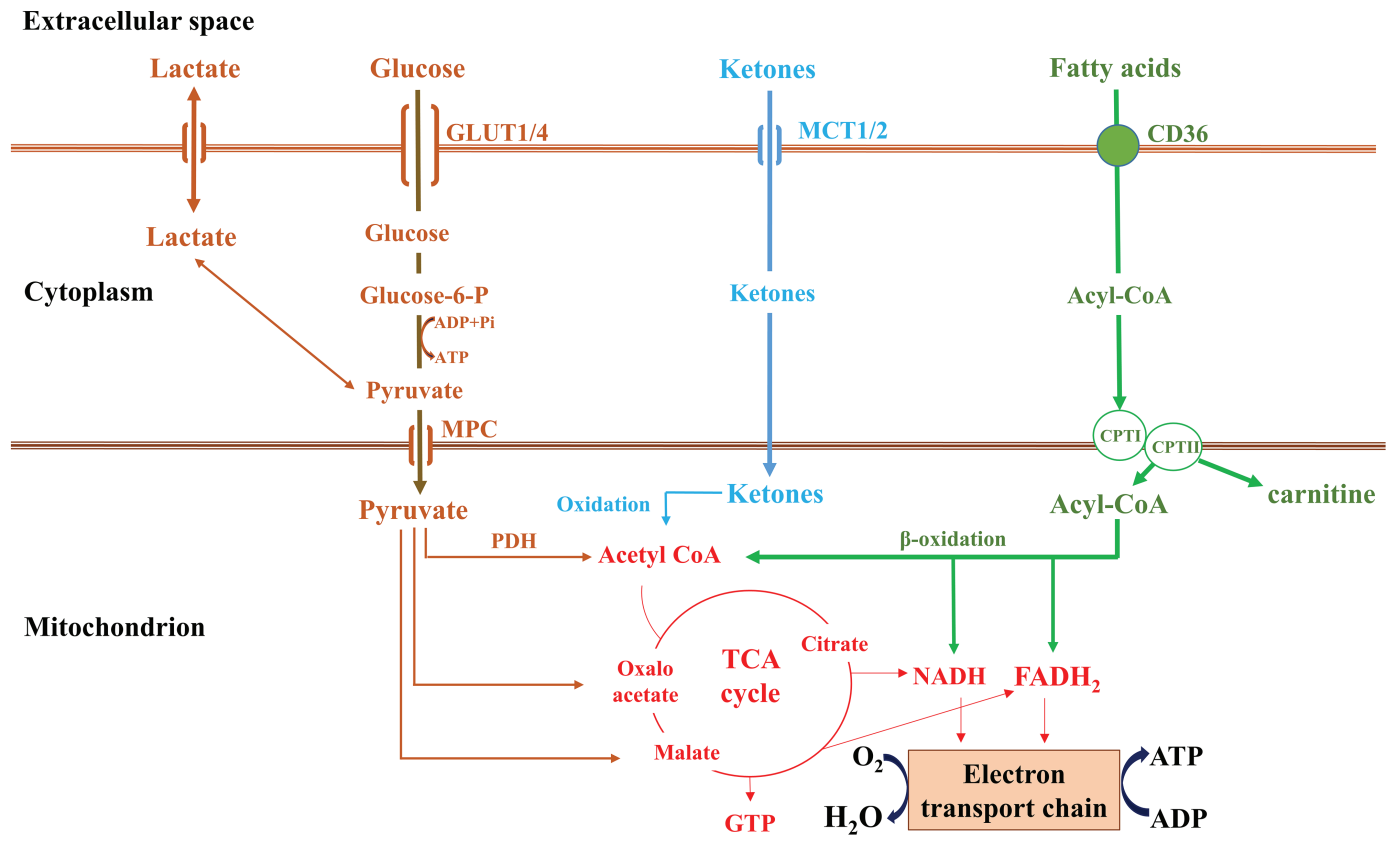
Metabolic pathways in normal heart.

Glucose is obtained by cardiomycoytes either from circulation or from hydrolysis of intracellular glycogen stores. Glucose serves as the main cardiomyocyte energy production fuel in the fetal hearts. Glucose uptake by cardiomyocytes occurs through insulin-dependent glucose transporters (GLUT4) or *via* the insulin-independent GLUT1. On entering the cell, glucose is phosphorylated by hexokinase to glucose-6-phosphate (G-6-P), which is used for glycolysis, glycogen synthesis, the hexosamine biosynthesis pathway, or the pentose phosphate pathway. Pyruvate produced during glycolysis is transferred *via* mitochondrial pyruvate carrier (MPC) to mitochondrial matrix, where it is converted to acetyl-CoA by pyruvate dehydrogenase (PDH), and enters the TCA cycle (Ma and Li, 2015). PDH complex (PDC) activity is inhibited by PDH kinases (PDKs) that respond to allosteric modifiers derived from glycolysis and FAO. Ketone bodies are produced in the liver during starvation and fasting. There are three types of ketone bodies that the heart uses: acetone, acetoacetate, and β-hydroxybutyrate (βOHB; Cotter et al., 2013). βOHB-dehydrogenase oxidizes βOHB to acetoacetate in mitochondria. Acetoacetate is then converted to acetoacetyl-CoA by the rate limiting enzyme succinyl-CoA:3-oxoacid-CoA-transferase (SCOT). Finally, acetoacetyl-CoA is converted into acetyl-CoA by acetyl-CoA acetyltransferase (Williams et al., 1971) and enters the TCA cycle and electron transport chain for generating ATP (Figure 1).

## Alterations in Lipid Metabolism During Cardiac Aging

Considering the heavy reliance of cardiac ATP synthesis on FAO, it is not surprising that either deprivation or oversupply of fatty acids can be detrimental (Noh et al., 2006). An imbalance between fatty acid uptake and oxidation in aged hearts leads to accumulation of medium-chain acyl-carnitines (Koves et al., 2008) and long-chain fatty acids that are incorporated in triglycerides, phospholipids, and other lipid subspecies, such as diacylglycerols and ceramides. Higher diacylglycerols and ceramides content has been associated with cardiac lipotoxicity, which has been observed in the elderly (Drosatos and Schulze, 2013), as well as in mouse models of cardiac aging along with lower cardiac FAO and higher serum non-esterified fatty acids (Kates et al., 2003; Rodriguez-Calvo et al., 2007; Wang et al., 2015).

Higher cardiac lipid content in aging may be sustained by increased expression of cardiac CD36, which mediates fatty acid transport (Koonen et al., 2007). In accordance, aged CD36-deficient mice have lower levels of intramyocardial lipids, higher mitochondria-derived ATP synthesis, and improved cardiac function compared with aged wild-type mice. While CD36 upregulation and fatty acid uptake seem to be detrimental for cardiac aging, no difference was detected in fasting-induced cardiac lipoprotein lipase activity of aged rats compared with young animals (Bergo et al., 1997). This indicates continuous release of lipoprotein-derived fatty acids in aged hearts, which in combination with higher CD36 expression, explains the increased cardiac lipid content in aging. However, neither inhibition nor activation of lipoprotein lipase offers significant therapeutic potential. Nevertheless, both deletion (Noh et al., 2006) and constitutive activation of lipoprotein lipase (Yagyu et al., 2003) result in cardiomyopathy, due to energetic failure and increased ceramide content (Park et al., 2008), Protein Kinase C signaling activation, and β-adrenergic receptor desensitization (Drosatos et al., 2011).

In humans, age-dependent decline in myocardial fatty acid utilization and ATP production has been observed (Kates et al., 2003). This finding correlates with lower expression of cardiac peroxisome proliferator-activated receptors (PPAR)α (Iemitsu et al., 2002), which is a central transcriptional regulator of proteins that are involved in cardiac energy metabolism (Pol et al., 2015). However, activation of PPARα does not seem to be a desirable intervention, due to similarities of constitutive cardiomyocyte-specific expression of PPARα with cardiomyopathy in diabetes (Finck et al., 2002). On the other hand, cardiomyocyte PPARγ constitutive expression is also toxic for the heart (Son et al., 2007), unless PPARα is inhibited (Son et al., 2010). Thus, as aged hearts have lower PPARα expression (Iemitsu et al., 2002); activation of PPARγ emerges as a potential therapeutic direction that warrants further investigation in models of cardiac aging. Thus the combination of increased lipid uptake and reduced lipid oxidation during cardiac aging may account for the observed cardiac lipotoxicity and associated complications.

## Carbohydrate Oxidative Metabolism in Aged Heart

Aged hearts have higher glycolysis and glucose catabolism at the expense of FAO (Lakatta and Yin, 1982). It is also noted that during aging, PDC phosphorylation by PDK is lower, which favors mitochondrial pyruvate oxidation (Moreau et al., 2004). Another study has also reported lower PDK4 expression in aged hearts compared with young hearts (Hyyti et al., 2010). The downregulation of PDK4 may be accounted for by the suppression of the expression of cardiac PPARα that has been reported in aging-associated cardiomyopathy (Iemitsu et al., 2002; Pol et al., 2015). Insulin resistance constitutes another layer of control of glucose utilization that pertains to glucose uptake by the tissues. In elderly, insulin resistance is reckoned as an independent risk factor for coronary heart disease (Butler et al., 2006). It is observed that IGF-1 deficiency enhances resistance of cardiomyocytes against aging (Li et al., 2008). Also, suppression of phosphoinositide 3-kinase (PI3K) weakens senescence and inflammatory markers expression and decreases in accumulation of the aging pigment and lipofuscin (Inuzuka et al., 2009). Thus, shift from FAO to glucose oxidation may constitute a compensatory action of cardiomyocytes aiming to balance the energetic deficiency caused by suppression of FAO. However, the increased glucose utilization in aged hearts and the alleviating effects of insulin signaling inhibition suggest that increased glucose utilization contributes in aging-related cardiomyopathy and may constitute a therapeutic target.

## Oxidation of Ketone Bodies in Aged Heart

Recent studies have suggested ketone bodies as an efficient alternative fuel for failing hearts (Takahara et al., 2021), which overall energy production without interfering with fatty acid or glucose catabolism (Ho et al., 2019). In light of the association of increased glucose catabolism with cardiac aging, ketone bodies oxidation emerges as a potential resource that may help alleviate aging-related cardiovascular complication. Adaptation from glycolysis to ketone oxidation is delayed in aged rats, which take longer time than young animals to produce high levels of circulating ketone bodies either when fed on ketogenic diet or following fasting (Okuda et al., 1987; Hernandez et al., 2018).

Ketone body production is increased by approximately 2-fold between 3 and 30 months of age in mice (Robinson and Williamson, 1980). Cardiac SCOT, which is a mitochondrial enzyme involved in the extrahepatic metabolism of ketone bodies, shows increased activity in aged rats due to higher nitro-hydroxyl modification of tryptophan 372 that is proximal to the active site (Rebrin et al., 2007). The beneficial metabolic effect of ketone bodies may rely on their antioxidant properties that pertain to the increased ratio between reduced and oxidized glutathione (Veech et al., 2001; Cahill and Veech, 2003) or their stimulatory effect on succinate oxidation (Balietti et al., 2009). The anti-oxidant effect of ketone bodies, which has been demonstrated by various studies (Mallet and Sun, 2003; Squires et al., 2003), may account for their protective effect in aged hearts as oxidative stress is one of the main manifestations of cardiac aging. The anti-oxidant effect of ketone bodies may also account for the improvements in mitochondrial repair mechanisms (Thai et al., 2019) or in mitophagy flux (Thai et al., 2019) in the aged hearts.

Nutritional interventions with cyclic ketogenic diet in old mice improve heart rate, fractional shortening, left aortic valve pressure gradient, and ventricular mass (Newman et al., 2017). At the same time, ketone bodies incur anti-oxidative and anti-inflammatory effects and counteract age-related cardiac remodeling (Shimazu et al., 2013; Puchalska and Crawford, 2017). Despite the beneficial metabolic effects of ketone bodies, it has been reported that they compete with fatty acids and inhibit their oxidation in diabetic hearts (Hasselbaink et al., 2003; Stanley et al., 2003), which results in lipid accumulation. Studies with isolated cardiomyocytes showed that chronic exposure to βOHB induces insulin resistance and suppresses glucose uptake and oxidation (Pelletier et al., 2007). Taking into account the association of glucose oxidation with cardiotoxicity in aging, ketone bodies hold promise for therapeutic interventions that aim to alleviate cardiomyopathy in the elderly.

## Mitochondrial Function in Cardiac Aging

Due to the high-energy demands of the heart, cardiomyocytes are rich in mitochondria, which account for ~35% of total cellular volume. Therefore, the heart is highly vulnerable to mitochondrial anomalies. There are two types of cardiac mitochondria with varying activities: the subsarcolemmal mitochondria (SSM) and the interfibrillar mitochondria (IFM) that are located between the myofibrils (Palmer et al., 1977). Aging primarily decreases content and function of IFM, associated with lower activity of mitochondrial respiratory chain complexes III and IV, impaired oxidative phosphorylation, and increased production of reactive oxygen species (ROS) that leads to injury (Fannin et al., 1999; Judge et al., 2005). Studies in humans and animals have reported that aged hearts have larger mitochondria with disrupted structure and inner membrane cristae (Sachs et al., 1977; Dai and Rabinovitch, 2009). Other studies have also reported lower content and activity of cytochrome oxidase in aged human hearts. This event has deleterious effect on cardiac energy production and exerts cytotoxicity (Fujioka et al., 2011).

Cardiac mitochondria of the elderly are more sensitive to lipid peroxidation and dysfunction when exposed to exogenous iron and H_2_O_2_ (Sawada and Carlson, 1987; Cusack et al., 1991). Aged mice have significantly lower expression of myocardial mitochondrial deacetylase sirtuin (SIRT)3, which counteracts oxidative stress. In young animals, nuclear factor erythroid-2 related factor (NRF-2), a highly conserved redox-sensitive transcription factor, is activated by ROS production and increases the expression of several antioxidant genes. However, in aging, ROS-dependent activation of NRF-2 is defective, which aggravates the deleterious effects of ROS (Warabi et al., 2007; Ungvari et al., 2011). Manganese superoxide dismutase, which transforms toxic superoxide to hydrogen peroxide and diatomic oxygen, thus preventing accumulation of mitochondrial ROS, is found to be reduced in aged hearts (Li et al., 2018). Along these lines, supplementation of polyunsaturated fatty acid-containing diet with the anti-oxidant coenzyme Q10 in 24-month old rats increased catalase activity, reduced H_2_O_2_ generation, improved cytochrome oxidase activity in cardiac mitochondria, and extended life span (Ochoa et al., 2005; Quiles et al., 2010).

In addition, mitochondrial protein carbonylation is increased with aging, which further aggravates mitochondrial oxidative damage (Dai et al., 2012). Mitochondrial DNA (mtDNA), which lacks protective histones, is particularly vulnerable to oxidative damage leading to point mutations and deletions, inflammation, apoptosis, telomere shortening, necrosis, and immunological dysfunction (Larsson, 2010; Steenman and Lande, 2017).

Besides oxidative stress, mitochondrial biogenesis is also compromised during cardiac aging. PGC-1α, which stimulates mitochondrial biogenesis, serves as a transcriptional coactivator of nuclear receptors, like PPARs, Estrogen-related receptor alpha (ERRα), nuclear factor erythroid-2 related factor (NRF)-1 and NRF-2 (Sanchis-Gomar et al., 2014; Dorn et al., 2015), and regulator of cardiac fatty acid utilization (Arany et al., 2005; Rowe et al., 2010). PGC-1α protein levels are lower in aged mice, compared to young mice (Conley et al., 2007; Vina et al., 2009) similar to what is observed in humans with heart failure (Sihag et al., 2009). PGC-1α knockout in young mice mimics age-related impairments in mitochondrial gene expression. On the other hand, overt activation of PGC-1α accelerates cardiac aging and reduces life span of old wild type mice because of mitochondrial damage and ROS accumulation (Zhu et al., 2019), indicating a delicate balance between PGC1α activation and inhibition for cardiac homeostasis. Indeed, moderate PGC-1α activation inhibits age-related cardiac remodeling changes, such as apoptosis and collagen accumulation, and increases the expression of genes involved in myocardial contractility, metabolism, biogenesis, dynamics, and calcium handling (Whitehead et al., 2018). In accordance with the genetic interventions, mild activators of SIRT1 and PGC-1α, such as resveratrol, incur beneficial effects in mitochondria of senescent HL-1 cardiomyocytes (Ren et al., 2017).

Mitochondrial function is regulated by cardiolipin, which is a main phospholipid of the inner mitochondrial membrane. Cardiolipin preserves the morphology and architecture of the mitochondrial membrane and regulates the activity of mitochondrial enzymes and proteins (Paradies et al., 2019). Some studies have attributed mitochondrial complications during aging to lower cardiolipin content that alters fluidity of the inner mitochondrial membrane. During oxidative stress, cardiolipin undergoes oxidation that disrupts the electron transport chain, leading to mitochondrial dysfunction, cytochrome c release, and cellular apoptosis (Paradies et al., 1998; Ott et al., 2002; Chicco and Sparagna, 2007).

Mitochondrial quality in cardiomyocytes is maintained through a balance between mitophagy and biogenesis. The mitochondrial defects that are observed during cardiac aging are further aggravated by impaired fission, fusion, autophagy, and lysosomal degradation. To correct flaws in mitochondrial function, there is a quality control process that includes biogenesis of new mitochondria and degradation or recycling of the old ones. Accumulation of dysfunctional mitochondria due to oxidative stress, mtDNA mutations, lowering of mitochondrial membrane potential, and defective ATP synthesis stimulate cellular apoptosis and premature cardiac aging. The frequency of mtDNA mutations in older mice is 1,000-fold higher than that for nuclear genes. Accordingly, human cardiac mtDNA deletions begin at the age of 40 years and accumulate gradually with age (Corral-Debrinski et al., 1992; Khaidakov et al., 2003).

Thus, reductions in mitochondrial number along with accumulation of defective mitochondria that constitute a source of ROS are major contributors of the pathophysiology of cardiac aging.

## Autophagy in Cardiac Aging

Autophagy is a highly conserved homeostatic mechanism in cells. It includes micro-autophagy, macro-autophagy, and chaperone-mediated autophagy and is regulated by a series of autophagy related genes (ATG). It is involved in degradation and recycling of cellular components, removal of misfolded proteins and damaged organelles, and internal “recycling” of nutrients (Rubinsztein et al., 2011). In cells with low proliferative capacity, like cardiomyocytes, accumulation of lethal proteins, and dysfunctional organelles is a critical event that impairs cellular homeostasis. As age advances the rate of autophagy and mitophagy decrease in heart as well as in other tissues (Hoshino et al., 2013; Xu et al., 2016). The decline in autophagy has a significant impact on the aging-associated accumulation of defective mitochondria and consequent ROS generation in cariomyocytes leading to mitochondrial dysfunction, cardiac aging, and myocardial injury (Mammucari and Rizzuto, 2010; Rezzani et al., 2012). Accordingly, autophagy is a vital mechanism for maintenance of homeostasis and adaptation to stress that the heart undergoes throughout the aging process (Levine and Kroemer, 2008). Lower autophagy that occurs during cardiac aging, results in the accumulation of protein aggregates, oxidized and damaged proteins, and lipofuscin (Dai et al., 2010). Lipofuscin is rich in iron and therefore, it causes lysosomal rupture, oxidative stress, and further mitochondrial damage (Brunk and Terman, 2002). Mouse models of diet-induced obesity with impaired autophagy have compromised myocardial geometry and function (Xu et al., 2013), which indicates that autophagy also alleviates cardiac lipotoxicity.

Protein carbonylation and ubiquitination in aged hearts along with reduced protein turnover rates (Basisty et al., 2018) compromise cardiac proteasome function that impairs protein quality control and leads to accumulation of damaged and misfolded proteins. It is also noted alteration in the expression of heat shock proteins (HSP), which function as molecular chaperones facilitating protein folding and targeting improperly folded proteins to degradative pathways are reduced in aged cardiomyocytes (Bodyak et al., 2002). Higher levels of ROS in aged cardiomyocytes stimulate formation of lipofuscin aggregates, consisting of lipids and aggregated liposomal proteins in lysosomes and which distort autophagy by preventing the fusion of autophagosomes with lysosomes leads to accumulation of aberrant proteins and dysfunctional mitochondria, less effective stress response, and functional loss in cardiomyocytes (Taneike et al., 2010).

Stimulation of autophagy improves cardiac function and prolongs the lifespan in many organisms (Miyamoto, 2019). Selective elimination of damaged mitochondria by autophagy (mitophagy) is an essential component of this mechanism. Rapamycin, which inhibits mTOR and induces autophagy, improves cardiac function and extends life span in C57BL/6 mice, which is also accompanied by increased mitochondrial biogenesis and fatty acid catabolism (Dai et al., 2014; Zhang et al., 2014; Quarles et al., 2020). Another intervention that activated autophagy, which rely on overexpression of ATG5, extended life span in mice. Accordingly, ATG5 deficiency in mouse hearts induces age-associated cardiomyopathy (Taneike et al., 2010; Pyo et al., 2013).

Beclin 1 regulates the initial stage of autophagy in mammalian cells by leading to the formation of a double-membrane structure that engulfs cytoplasmic material to form autophagosomes. Bcl-2 is inhibiting activity of beclin 1 by directly binding to it (Qu et al., 2003; Pattingre et al., 2005). Disruption of the Beclin1-Bcl2 complex stimulates autophagy and inhibits age-induced cardiac fibrosis, hypertrophy, apoptotic cell death, and delays cardiac aging (Fernandez et al., 2018).

Parkin is an E3 ligase that mediates selective identification and removal of damaged mitochondria by autophagy. Parkin-deficient mice accumulate unusual mitochondria in the heart as they age, while parkin transgenic mice display increased mitophagy and are resistant to cardiac aging (Hoshino et al., 2013; Kubli et al., 2013).

The mitochondrial membrane depolarization-dependent PTEN-induced putative kinase (PINK)1/Parkin pathway is involved in tagging defective mitochondria, which is vital for maintaining cardiac function during aging (Nguyen et al., 2016). Autophagy is controlled by various metabolism-related pathways, including the IGF-1/Akt pathway (Ock et al., 2016), the mammalian target of rapamycin (mTORC1) pathway (Harrison et al., 2009), the AMP-activated protein kinase (AMPK) pathway (Kim et al., 2011), Forkhead box O (FoxO), the transcription factor EB (TFEB) pathway (Fullgrabe et al., 2016), ROS (Shirakabe et al., 2016), and sirtuins (Alcendor et al., 2007).

Thus, aging-related cardiomyopathy is associated with lower autophagic flux. Hence, autophagy is a promising therapeutic target for cardiac aging.

## Metabolic Deficiency and Inflammation in Cardiac Aging

A close link between cellular oxidative stress and inflammation exists and contributes in the cardiac aging process (De La Fuente, 2002). Inflammation is stimulated by accumulation of senescent cardiomyocytes that leads to senescence-associated secretory phenotype (SASP). GDF15, Tgfb2, and Edn3 are nontypical SASP that are secreted by cardiomyocytes. Chronic stimulation of the immune system attenuates immune response, which is typical for inflammation-associated aging (“inflammaging”; Douziech et al., 2002) that is controlled by NF-κB (Salminen et al., 2008a). Inhibition of aging-related NF-κB activation by the longevity gene, SIRT1 delays aging (Salminen et al., 2008b; Cevenini et al., 2013). Circulating levels of activin, a senescence-associated secreted protein has been correlated with age-related heart failure (Li et al., 2020). Thus, defects in the metabolic machinery of aged cardiomyocytes exert pro-inflammatory effects that compromise cardiac function further.

## Epilogue

**Figure 2:**
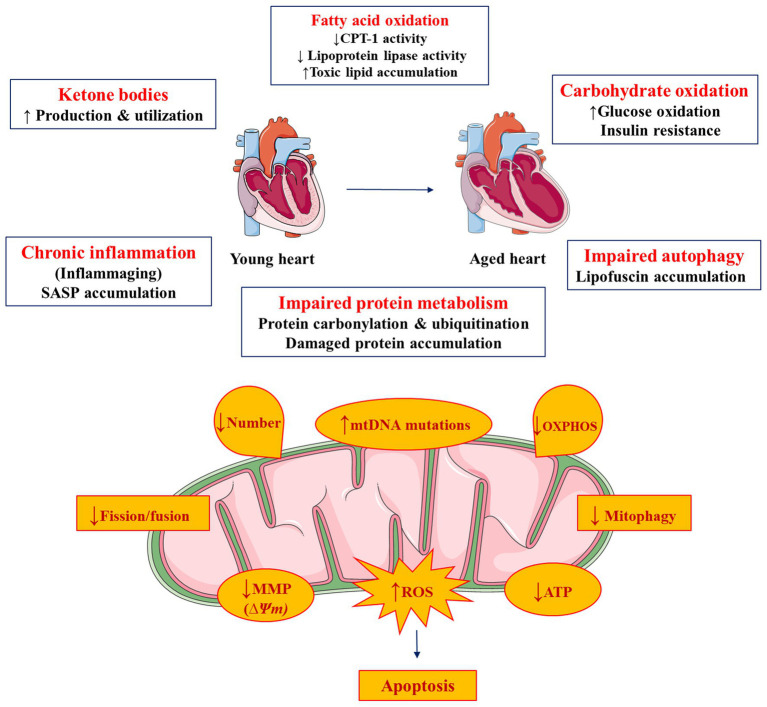
Metabolic and mitochondrial complications in cardiac aging. Figure was produced using Servier medical art (http://www.servier.com).

Heart is an organ with high energetic demands that are achieved through oxidation of various substrates. Utilization of fatty acids, which is the major substrate for energy production, is reduced during cardiac aging at the expense of higher carbohydrate oxidation. This leads to accumulation of toxic lipids in the heart. These events are associated with mitochondrial dysfunction and eventual accumulation of ROS that further aggravate cardiac dysfunction. The higher inflammatory status and impaired autophagy compromise reparative processes that could further worsen aging-related cardiomyopathy (Figure 2).

The variety of metabolic pathways that are affected during cardiac aging suggests potential novel therapeutic interventions. Particularly, interventions that aim to negate aging-induced defects in mitochondria and intermediate metabolism are warranted. Based on clinical observations and preclinical interventions, treatments that will stimulate oxidation of fatty acids and ketone bodies, while they will simultaneously limit glucose catabolism, maintain normal autophagic flux, and limit mitochondrial ROS accumulation hold significant therapeutic potential. Thus, additional studies aiming to elucidate metabolic changes that occur during the progression of cardiac aging are warranted, in order to identify novel therapeutic avenues that may eventually translate to treatments of aging-related cardiovascular complications in humans.

## Author Contributions

TS reviewed the literature and wrote the manuscript. KD reviewed the literature and edited the manuscript. All authors contributed to the article and approved the submitted version.

### Conflict of Interest

The authors declare that the research was conducted in the absence of any commercial or financial relationships that could be construed as a potential conflict of interest.
